# Predictive Modeling of Electric Bicycle Battery Performance: Integrating Real-Time Sensor Data and Machine Learning Techniques

**DOI:** 10.3390/s25051392

**Published:** 2025-02-25

**Authors:** Catherine Rincón-Maya, Daniel Acosta-González, Fernando Guevara-Carazas, Freddy Hernández-Barajas, Carmen Patino-Rodríguez, Olga Usuga-Manco

**Affiliations:** 1 Departamento de Ingeniería Industrial, Universidad de Antioquia, Medellín 050010, Colombia; catherine.rincon@udea.edu.co (C.R.-M.); elena.patino@udea.edu.co (C.P.-R.); 2 Departamento de Ingeniería de Sistemas, Universidad de Antioquia, Medellín 050010, Colombia; dfernando.acosta@udea.edu.co; 3 Departamento de Ingeniería Mecánica, Universidad Nacional de Colombia Sede Medellín, Medellín 050034, Colombia; fjguevarac@unal.edu.co; 4 Departamento de Estadística, Universidad Nacional de Colombia Sede Medellín, Medellín 050034, Colombia; fhernanb@unal.edu.co

**Keywords:** machine learning, remaining useful life, data-driven models, lithium ion battery

## Abstract

In the field of sustainable mobility, this study highlights the importance of using machine learning for predictive modeling based on real traffic data collected from instrumented bicycles. The advent of advanced technologies like sustainable mobility apps, sensors, and advanced data analysis methods led to the ability to collect data from various sources, which enabled researchers to estimate battery state of charge (SOC) accurately. Most current research uses them in the lab experiments for data collection. In this work, we use real-time sensors data to construct data-driven models for lithium-ion battery SOC estimation. This research integrates both electric bicycle battery, environmental and route variables to achieve the following goals: (1) Collect a multimodal data set including operational, topography, vehicle, and external variables, (2) Preprocess data obtained from sensors installed on the electric bicycle battery, (3) Create models of lithium-ion battery SOC based on electric bicycle battery and environmental variables, and (4) Assess data-driven models and compare their performance for lithium-ion battery SOC with high accuracy. To achieve that, we conducted a real study to predict the Remaining Useful Life (RUL), as a measure of state of charge, of electric bicycle battery. The study was carried out on a 15 km cycle route in Medellín, Colombia, for 28 days. To estimate the RUL, we used four different machine learning algorithms: Long Short-Term Memory (LSTM), Support Vector Regression (SVR), AdaBoost, and Gradient Boost. Notably, data preprocessing techniques played a pivotal role, with a particular focus on smoothing sensor data using Convolutional Neural Networks (CNN). The results showed a significant improvement in prediction accuracy when using data preprocessing, confirming its importance in improving model performance. Furthermore, the comparison of network performance facilitated the selection of the most effective model for the test data. This study underscores the value of using real-world data to develop and validate predictive models in the pursuit of sustainable mobility solutions, and highlights the critical role of data-driven methodologies in addressing today’s urban transportation challenges.

## 1. Introduction

The transportation sector on a global scale is a major contributor to pollution, carbon emissions, and dependence on finite fossil fuels [1,2,3,4]. In response, governments are implementing policies to enhance energy security, reduce emissions, and promote sustainable mobility [1,4,5]. The rapid expansion of the electric vehicles (EVs) market underscores this transition, with projections that EVs will account for approximately 57% of global vehicle sales by 2040 [3]. However, a key challenge remains the development of predictive failure analysis systems to optimize vehicle performance and lifetime [1,3]. Electromobility integrates advanced transportation technologies, including small EVs that are lightweight, energy efficient, and cost effective [6]. These vehicles rely on critical components, primarily the battery and motor, with battery costs accounting for nearly 30% of the total vehicle cost [2,5,7]. Consequently, battery performance is critical to ensuring the reliability, efficiency, and sustainability of EVs [8]. Predictive models have been developed primarily using publicly available data sets or laboratory-controlled experiments, which are often costly due to the equipment, time and personnel required [5,9,10,11]. However, these studies do not account for real-world variables such as traffic conditions, environmental factors, and topography. In contrast, ref. [12] conducted field experiments that included these external factors and identified key parameters that influence the EVs’ performance. Field studies provide valuable insight into the relationships between variables under uncontrolled conditions, improving the accuracy and applicability of the model.

Critical components of small EVs require a robust safety and reliability management system to ensure optimal performance [5]. Safety measures focus on protecting occupants, pedestrians and the environment, while reliability ensures that small EVs perform their intended functions consistently over time. Among these components, the battery management system (BMS) plays a critical role in overseeing battery performance, safety, and longevity by monitoring key parameters such as state of charge, temperature, and cell balance. Accurately estimating the State of Health (SOH) and predicting the Remaining Useful Life (RUL) are essential for assessing battery performance and ensuring the safe and reliable operation of small EVs [2,5]. Given the role of the battery as a critical power source, understanding its degradation patterns and potential failures is critical to maintaining operational efficiency, minimizing unexpected downtime, and optimizing maintenance strategies. Integrating advanced monitoring and predictive maintenance techniques can improve EV safety and extend battery RUL.

SOH represents the overall health of a system or component, specifically the loss of battery capacity due to aging, usage patterns and environmental conditions. RUL, on the other hand, estimates the number of charge–discharge cycles remaining before performance degradation renders the battery ineffective. One of the major challenges in managing critical EV components is the inherent complexity of degradation processes, which involve physicochemical reactions and nonlinear parameter variations over time [2]. Without proper monitoring, battery degradation can lead to serious safety hazards such as thermal runaway, leakage, and explosion. Therefore, real-time monitoring of degradation parameters is essential to mitigate these risks. Accurately predicting RUL under real-world operating conditions enables proactive maintenance strategies, reducing the likelihood of sudden failures and improving EV reliability [2,5]. As EV adoption continues to grow, improving SOH estimation and RUL prediction methods remains a priority to ensure sustainable and safe electric mobility.

Predictive models in the context of EVs play a critical role in optimizing performance, improving operational efficiency, and enhancing safety. These models use large data sets to analyze complex interactions between vehicle components and external conditions, enabling accurate estimation of key parameters such as energy consumption, thermal behavior, and mechanical wear. Unlike traditional rule-based approaches, predictive models incorporate machine learning and statistical techniques to dynamically adjust predictions based on real-time and historical data [12]. This adaptability is especially critical for EVs operating in diverse environments where temperature variations, road conditions, and driving behavior introduce significant variability. In addition, predictive modeling enhances the development of intelligent BMS by improving SOH and RUL estimates, enabling proactive maintenance strategies that minimize downtime and extend battery life. By integrating predictive analytics with real-world operational data, these models drive autonomous diagnostics and intelligent decision-making, ultimately ensuring the long-term reliability and sustainability of EVs.

Prognostics and health management (PHM) techniques for Li-ion batteries are classified into physics of failure (PoF), data-driven, and hybrid approaches, each with distinct advantages and limitations. PoF-based models rely on fundamental physical and chemical principles to describe battery degradation, requiring a deep understanding of electrochemical reactions, material properties, and impedance variations. Despite their accuracy, these models are computationally expensive and unsuitable for real-time applications due to their reliance on complex mathematical formulations [9,13,14]. In contrast, data-driven methods use statistical algorithms and machine learning techniques to predict the RUL of batteries without the need for explicit physical modeling. These methods analyze degradation data collected over long periods of time to identify patterns and correlations. Although data-driven approaches require large datasets and computational resources, their adaptability and efficiency have made them widely applicable for SOH estimation and RUL prediction, especially in scenarios where real-time processing and computational efficiency are required [5,9,15].

While hybrid models integrate PoF and data-driven techniques have improved prediction accuracy and computational efficiency, most existing approaches primarily focus on controlled laboratory conditions or general urban environments. Although time series models, Filter-based techniques, including Kalman Filters (KF) and Particle Filters (PF), have been effective in tracking SOH and predicting battery degradation, they often fail to incorporate the influence of external variables such as altitude, temperature fluctuations, and road gradients [2,5]. To improve RUL estimation and real-time applicability, recent advances have sought to enhance these models by integrating deep learning and probabilistic methods [10]. However, despite these improvements, current methodologies rarely consider the extreme environmental and operational conditions that affect battery longevity in challenging terrains.

The Andean region presents unique topographical and environmental challenges that significantly impact the performance and longevity of EV batteries. High altitude conditions expose batteries to low temperatures and reduced oxygen levels, increasing internal resistance and affecting lithium-ion mobility, leading to inaccuracies in state of charge (SOC) and SOH estimates [16,17]. In addition, steep gradients demand higher energy output, accelerating battery degradation, while prolonged downhill driving reduces the effectiveness of regenerative braking, limiting energy recovery [18,19]. Despite the growing adoption of EVs in countries such as Colombia, where over 800,000 motorcycles are expected to be sold by 2024, research integrating topographical, operational, and environmental factors into predictive models remains limited [20].

To address this gap, this study makes a significant contribution by the collecting a multimodal dataset that includes real-world data from EVs operating in Medellín, Colombia, capturing variations in energy consumption, battery degradation, and external environmental conditions. In addition, this research applies advanced data preprocessing techniques to improve the accuracy and reliability of machine learning models used for battery diagnostics. This multifaceted approach summarizes the follow contributions: (1) Preprocessing data obtained from sensors installed on the electric bicycle battery to improve predictive accuracy. In addition, the study develops predictive models specifically designed to estimate RUL under the challenging conditions of high altitude terrain; (2) Creation of models of lithium-ion battery on electric bicycle battery performance and environmental variables; (3) Assessing data-driven models and comparing their performance for lithium-ion battery SOC with high accuracy. By integrating these methods, the research provides valuable insights for optimizing EV battery performance, improving predictive maintenance strategies, and enhancing sustainable mobility in mountainous regions.

The rest of the article is organized as follows. Section 2 covers previous work related to RUL prediction methods. The materials and methods used are discussed in Section 3 including data collection, machine learning methods, method for preprocessing and measures to evaluate the performance. Section 4 presents the results of RUL models and compares their performance. Finally, Section 5 and Section 6 present the discussions and conclusions of the work.

## 2. Related Work

This section presents previous research related to preprocessing and RUL prediction methods.

### 2.1. Previous Research on Preprocessing Methods

Convolutional Neural Networks (CNN) have emerged as powerful tools for preprocessing and feature extraction in RUL and SOH estimation, offering significant advantages over traditional methods. Unlike autoregressive models (AR, ARMA, ARIMA), KF, and PF, CNNs can automatically learn hierarchical features from raw data, eliminating the need for manual feature engineering [2,5]. Traditional machine learning techniques such as Support Vector Machines (SVM), Gaussian Process Regression (GPR), and Fuzzy Logic (FL) require extensive domain knowledge to extract relevant features for degradation analysis [9,14]. Moreover, while Kalman-based methods such as the Extended Kalman Filter (EKF) and Unscented Kalman Filter (UKF) can model nonlinearities in lithium-ion battery degradation, they rely on precise system parameters and are susceptible to divergence under uncertain conditions [14]. CNN, by contrast, effectively model complex relationships in high-dimensional data while demonstrating robustness against noisy sensor inputs, a major challenge for traditional filtering techniques [2,5].

CNN demonstrates a notable advantage in its capacity to automate feature extraction, thereby markedly diminishing its reliance on predefined degradation indicators, including impedance variations and electrochemical state estimations. Traditional approaches often require extensive preprocessing and handcrafted features based on electrochemical principles, whereas CNN autonomously identify meaningful patterns from raw sensor data, enhancing prediction accuracy [9,13]. Furthermore, CNN excel in handling the nonlinear degradation behavior of lithium-ion batteries, which remains a significant limitation for model-based techniques. While KF can track state variables in dynamic systems, they require extensive tuning and struggle with uncertainties in degradation processes [14]. Similarly, PF demand a high number of particles to ensure accuracy, which increases computational costs. CNN mitigate these challenges by leveraging convolutional layers to extract relevant degradation features while filtering out irrelevant variations, thereby improving predictive performance [2,5].

Beyond feature extraction and noise reduction, CNN offer computational efficiency in large-scale data processing, making them well-suited for real-time applications. Traditional statistical methods, such as ARIMA, process data sequentially, limiting scalability in high-dimensional datasets. In contrast, CNN exploit parallelized computations, enabling rapid feature extraction from vast data streams [9,21]. Additionally, CNN can be integrated with Long Short-Term Memory (LSTM) architectures, to further enhance RUL estimation accuracy. These hybrid models capture both spatial and temporal dependencies, overcoming limitations associated with single-model approaches [13,15,22]. The adaptability of CNN models to varying operational conditions makes them a preferred choice for advanced BMS, ultimately improving predictive maintenance strategies and extending battery lifespan in electric vehicles operating under complex environmental conditions [23,24].

### 2.2. Previous Research on RUL Prediction Methods

Obtaining accurate RUL estimations of lithium-ion batteries is critical to ensuring the reliability and safety of EVs. Various predictive modeling approaches have been developed to assess battery degradation, including physics-based models, hybrid approaches, and data-driven techniques. Among these, data-driven methods have gained significant attention due to their ability to handle complex degradation patterns without requiring explicit physical modeling [2,9]. These models use historical and real-time sensor data to extract patterns and correlations that enable robust RUL estimation under varying operating conditions. Among data-driven methods, machine learning algorithms have emerged as powerful tools for RUL prediction, offering high adaptability and accuracy. These algorithms can be categorized into three main types: deep learning, traditional machine learning, and ensemble methods. Each category uses different computational strategies and optimization techniques to improve prediction accuracy. The following sections outline the characteristics and applications of these approaches, emphasizing their role in advancing battery health monitoring and predictive maintenance strategies.

Hybrid methods leverage the strengths of combining multiple algorithms to enhance predictive performance, often by compensating for the weaknesses of individual models. These approaches aim to create more robust models by merging the capabilities of different types of algorithms. For example, LSTM-AdaBoost combines the power of LSTM networks with the boosting technique of AdaBoost, which iteratively improves the accuracy of weak learners through weighted updates. Similarly, the CNN-XGBoost hybrid integrates CNN with XGBoost, a highly efficient implementation of Gradient Boosting that excels at handling structured data and providing accurate predictions. Another notable hybrid is CNN-LSTM-ASAN, which integrates CNN for feature extraction, LSTM for modeling sequential dependencies, and the Adaptive Sparse Attention Network (ASAN), which enhances training efficiency by focusing attention on the most informative features and minimizing redundant information Table 1 summarizes various predictive models used in estimating RUL for EVs, highlighting both the algorithms used and their corresponding performance metrics. The models are categorized into two main groups: data-driven methods and hybrid methods.

In terms of performance metrics, several data-driven algorithms demonstrate notably low mean square error (RMSE) values, indicating high predictive accuracy. The LSTM model stands out with an RMSE of 0.0036, highlighting its exceptional precision in predicting RUL. Similarly, Support Vector Regression (SVR) achieves an RMSE of 0.0132, further affirming the effectiveness of advanced regression techniques in this context. Among ensemble methods, Gradient Boosting and AdaBoost deliver competitive RMSE values of 0.0136 and 0.0274, respectively, demonstrating their strong predictive capabilities. In the case of hybrid approaches, both CNN-LSTM-ASAN and CNN-XGBoost exhibit solid performance, with RMSE values of 0.0212 and 0.0100, respectively, suggesting that combining different models can indeed enhance prediction accuracy.

Data-driven methods, like LSTM and SVR, offer strong predictive performance with low RMSE values. Their simplicity makes them easier to implement, maintain, and understand, which is valuable for practical applications. This transparency helps stakeholders trust and use the predictions effectively. In contrast, hybrid models, while potentially more accurate, are often complex. Combining multiple algorithms can make these models harder to design, implement, and interpret, which may limit their usability in real-world scenarios. In summary, although hybrid methods might provide slight accuracy improvements, data-driven approaches stand out due to their combination of high accuracy, simplicity, and interpretability. These characteristics make them better suited for predicting RUL in EVs, ensuring a practical and efficient solution that can be easily adopted in industrial environments.

## 3. Materials and Methods

This section outlines our approach to lithium-ion battery state of charge estimation. We begin by describing our data collection process, detailing the sources and scope of the information gathered. Next, we explain the machine learning methods employed to extract insights from this data. We then introduce the CC-CNN (Control Chart and a Convolutional Neural Network) method used for preprocessing the raw data to enhance feature extraction. Finally, we present the measures used to evaluate the performance of our models and validate our findings. These subsections provide a comprehensive overview of our methodological framework, ensuring reproducibility and transparency in our research.

### 3.1. Data Collection

**Figure 1:**
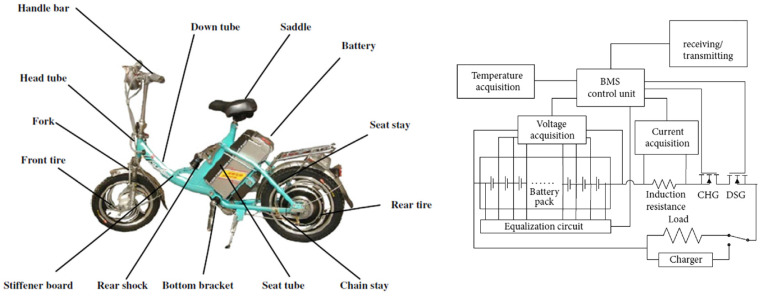
Small EVs used in tests.

The proposed analysis uses small EVs that, despite their smaller size, rely on battery banks as their primary energy source, mirroring the functionality of larger EVs. Figure 1 shows the small EVs used in tests, which was evaluated in its factory condition. These tests verified manufacturer-specified operating values and provided insight into key performance metrics such as speed and distance traveled. The sensor selection for instrumentation was based on Failure Mode Effects and Criticality Analysis (FMECA) results.

**Figure 2:**
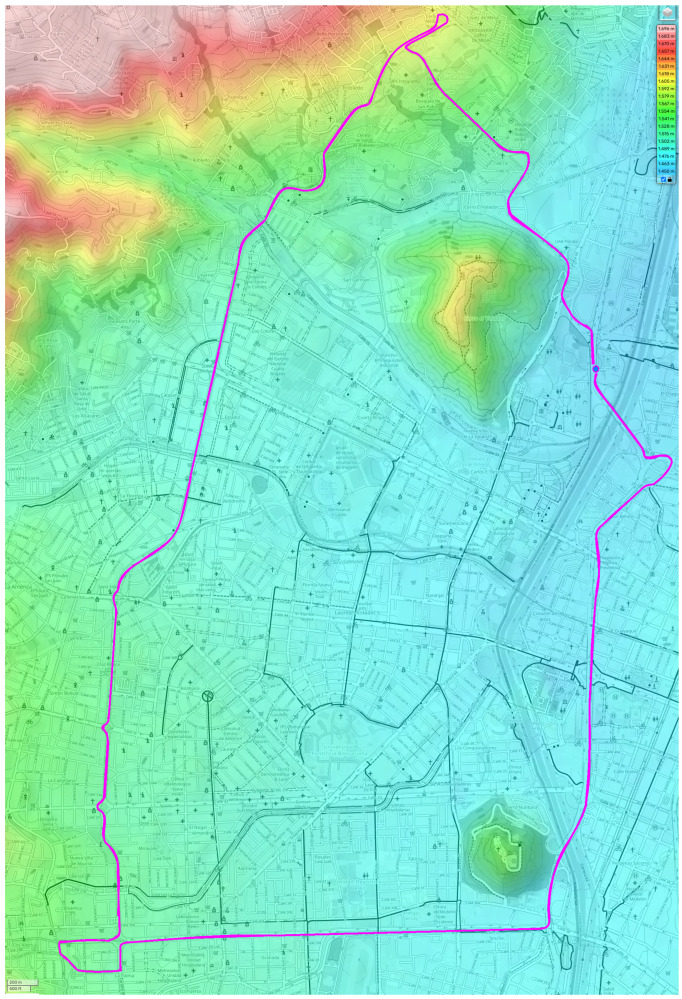
Route used in tests.

The data collection methodology was carefully designed to ensure controlled yet challenging test conditions. The predefined route included steep grades and high acceleration requirements, with all tests performed by the same driver to eliminate variability due to driver behavior. This methodology allowed an accurate assessment of battery performance under extreme operating conditions, including high-stress wear and tear at altitudes above 1450 m above sea level, as observed in the Andean region. Road tests were conducted in Medellín, Colombia, under urban driving conditions (see Figure 2).

Data collection included speed variations, distance traveled, altitude changes, and thermal monitoring of critical components such as the electric motor and battery. The vehicles were intentionally driven to catastrophic failure, a deliberate approach to accelerate battery degradation and assess performance under real-world stress scenarios. The experimental tests conducted along the predefined route using the specified instrumentation, generated 71,839 observations across 19 variables. These tests were conducted over multiple charge and discharge cycles, pushing the vehicles to their operating limits to capture critical data on battery degradation and overall performance. This comprehensive data set serves as a robust foundation for predictive modeling and analysis. Table 2 shows the averages of input variables related to route conditions and battery load.

Instrumentation sensors were selected following FMECA outcomes, preliminary test results, thermal captures, literature review, and a detailed analysis of electrical and electronic components (see Table 3).

Table 3 details the variables to measure, selected sensors, quantity for installation, and precision or tolerance of each. Sensor selection is based on measurement accuracy, size, and weight to accommodate installation spaces in the battery, motor, and controller. Additionally, sensor measurement ranges cover values observed during EV tests, minimizing measurement errors.

**Figure 3:**
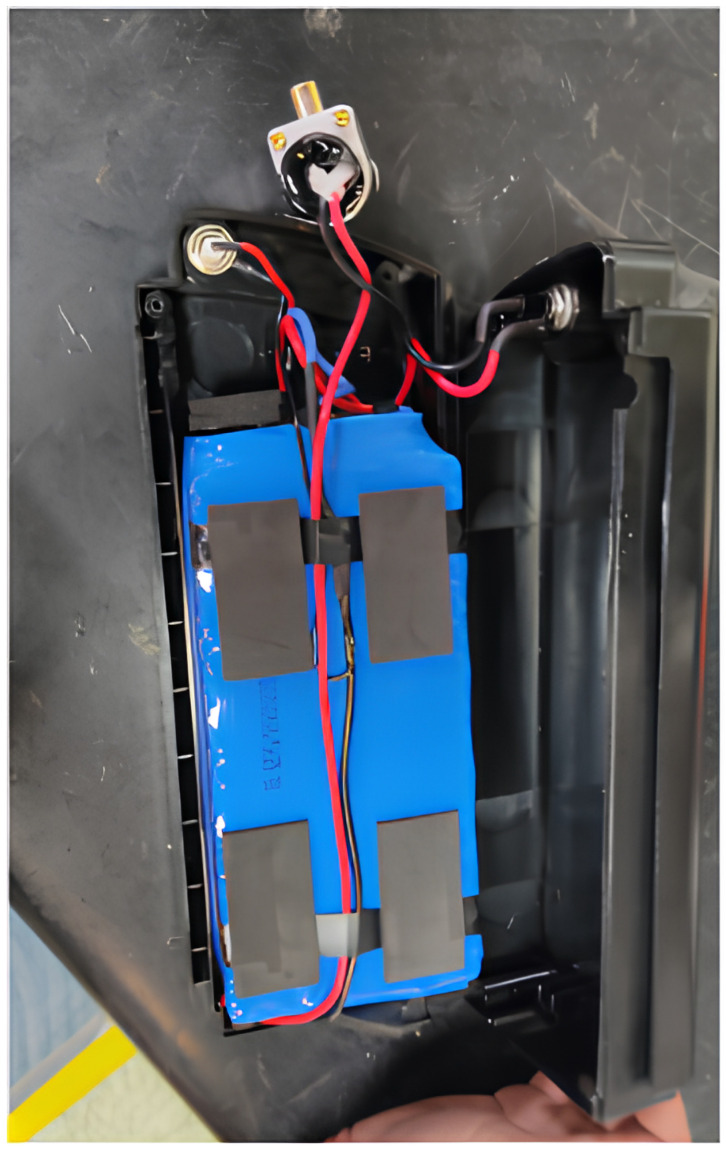
Installation of temperature sensors in EV batteries.

Temperature sensor positioning was determined considering battery temperature differentials, with two points on the battery and motor winding for accurate measurement. Figure 3 shows the installation of temperature sensors in EV batteries. The controller temperature will be measured on its casing to avoid invasive components, mitigating potential failure risks.

### 3.2. Machine Learning Methods

This section outlines the machine learning algorithms used in this study. SVR, introduced by [37], is derived from the SVM framework proposed by [38]. The formulation of SVR adheres to the principle of structural risk minimization, a concept established by SVM that has been shown to outperform the conventional empirical risk minimization approach commonly used in traditional machine learning techniques [39]. The primary objective of SVR is to define a tolerance range within which data points are expected to fall, both inside and outside the defined bounds. The optimization process focuses only on the observations that fall outside these bounds, constructing an objective function that depends on variable coefficients, prediction errors, and a cost parameter. The minimization of this objective function facilitates the determination of the optimal SVR coefficients, thereby improving the generalization ability of the model and the accuracy of its predictions.

AdaBoost (adaptive boosting) was proposed by [40]. It involves creating several short trees (stumps) sequentially, such that each subsequent tree adjusts well what the previous one could not. This ensemble approach progressively enhances the model’s performance, resulting in a final model that surpasses any individual weak regressor. AdaBoost is attractive due to its simplicity of use and less chance of overfitting problems compared to learning techniques [41].

Gradient Boosting was introduced by [42,43] and involves creating a sequence of predictors. The initial predictor utilizes the mean of the response variable for prediction, while subsequent predictors are built to correct the errors of their predecessors. This process continues iteratively, with each predictor refining the model by addressing the errors left by the previous one. This process can produce robust and interpretable models for both regression and classification [44].

LSTM represent a distinct variant of recurrent neural networks (RNN) with advanced deep learning capabilities, initially introduced by [45], specifically tailored to capture long-term dependency information [15]. LSTM architecture comprises a collection of interconnected subnetworks, referred to as memory cells, which retain their states over time and manage the flow of information via nonlinear activation units [46].

The following subsections show the details of the machine learning methods used in the article.

### 3.3. SVR

SVR is a powerful supervised learning algorithm derived from SVM framework, traditionally used for classification tasks. The goal of SVR is to find a function f(x) that has at most ϵ deviation from the actual target values *y* for a given dataset, while also maintaining a trade-off between the complexity of the model and the accuracy of prediction. This is achieved by minimizing a cost function subject to constraints on prediction errors.

Given a training dataset ${\left\{\left(x_{i},y_{i}\right)\right\}}_{i=1}^{n}$, where $x_{i}\in R^{d}$ represents the input features and $y_{i}\in R$ represents the target values, SVR seeks to find a linear function:

$$f\left(x\right)=w^{⊤}x+b,$$

where $w\in R^{d}$ is the weight vector and b∈R is the bias term. The optimization problem for SVR can be expressed as:

$$\min_{w,b,\xi ,\xi^{*}}\frac{1}{2}{∥w∥}^{2}+C\sum_{i=1}^{m}\left(\xi_{i}+\xi_{i}^{*}\right)$$

subject to:

$$\left\{\begin{array}{c} y_{i}-w^{⊤}x_{i}-b\leq \epsilon +\xi_{i}\\ w^{⊤}x_{i}+b-y_{i}\leq \epsilon +\xi_{i}^{*}\\ \xi_{i},\xi_{i}^{*}\geq 0 \end{array}\right.$$

**Figure 4:**
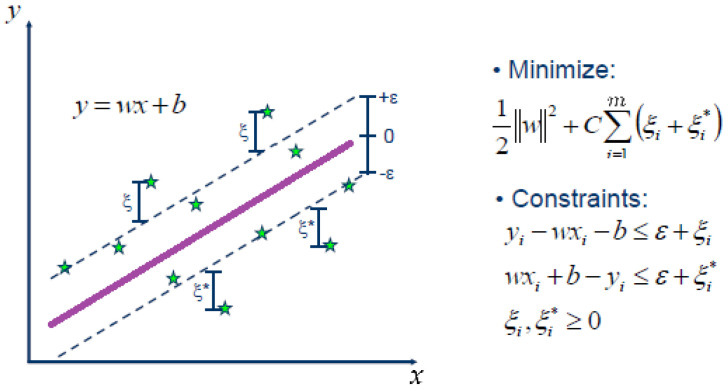
Illustration of SVR. Taken from: https://www.saedsayad.com/support_vector_machine_reg.htm (accessed on 22 December 2024).

Figure 4 illustrates the SVR for only one predictor *x*. Green stars represent the *n* observations, and the objective is to determine the purple line with slope *w*, an intercept *b* and a deviation ϵ to create a margin with *m* green stars outside that are used to estimate the line parameters.

In general, ϵ is a tolerance parameter that defines a tolerance margin within which the predictions are considered acceptable. The quantities $\xi_{i}$ and $\xi_{i}^{*}$ are slack variables that allow for violations of the ϵ-margin. The *C* is a regularization parameter that controls the trade-off between model complexity (flatness of *f*) and the amount by which deviations larger than ϵ are tolerated.

For nonlinear relationships, the SVR can be extended by mapping the input features to a high-dimensional space using a kernel function $K(x_{i},x_{j})$. The resulting optimization problem becomes the following.

$$f\left(x\right)=\sum_{i=1}^{m}\left(\alpha_{i}-\alpha_{i}^{*}\right)K\left(x_{i},x\right)+b,$$

where $\alpha_{i}$ and $\alpha_{i}^{*}$ are Lagrange multipliers. The more common kernel functions are listed below:

- Linear Kernel: $K\left(x_{i},x_{j}\right)=x_{i}^{⊤}x_{j}$.
- Polynomial Kernel: $K\left(x_{i},x_{j}\right)={\left(\gamma x_{i}^{⊤}x_{j}+r\right)}^{d}$.
- Radial Basis Function (RBF) Kernel: $K\left(x_{i},x_{j}\right)=\exp(-\gamma ∥x_{i}-x_{j}{∥}^{2})$.

SVR is particularly useful in scenarios where the goal is to predict continuous values with robustness to outliers. Its flexibility through kernel functions makes it capable of modeling complex nonlinear relationships effectively.

### 3.4. AdaBoost

AdaBoost is a powerful ensemble learning technique that is primarily used for classification tasks. The algorithm was introduced by Yoav Freund and Robert Schapire in [40] and is designed to improve the accuracy of weak classifiers by combining them into a single strong classifier.

AdaBoost can also be adapted for regression problems through a variant known as AdaBoost.RT (Regression Trees) proposed by [47]. In this context, instead of classifying instances into discrete categories, AdaBoost.RT predicts continuous outcomes by minimizing the mean squared error between predicted and actual values. This adaptation involves assigning weights based on the errors of previous predictions and updating them iteratively to refine the model’s accuracy. The core concept of AdaBoost.RT introduces a constant ϕ as a relative error threshold that distinguishes between correct and incorrect predictions. The error rate is then determined by tallying the number of correct and incorrect predictions. As a result, a weight updating parameter is calculated, and the distribution of the training set is modified based on this weight updating parameter [47].

The steps for AdaBoost.RT are described below.

1. Input:
  - Sequence of *n* examples ${\left\{\left(x_{i},y_{i}\right)\right\}}_{i=1}^{n}$, where $x_{i}\in R^{d}$ represents the input features and $y_{i}\in R$ represents the target values.
  - Select the weak learning algorithm.
  - Specify the integer *T* for the number of iterations.
  - Define the threshold ϕ to delimit the correct and incorrect predictions.
2. Initialize:
  - Start iteration t=1.
  - Define the first distribution as $D_{t}\left(i\right)=1/n$ for all *i*.
3. Iterate: while t≤T
  - Call the weak learner providing it with distribution $D_{t}$.
  - Build the regression model: $f_{t}\left(x\right)\to y$.
  - Calculate the error rate of $f_{t}\left(x\right)$ as $\epsilon_{t}=\sum_{i=1}^{n}D_{t}\left(i\right)$ where the value *i* is defined as $i:=\left|\frac{f_{t}\left(x_{i}\right)-y_{i}}{y_{i}}\right|>\phi$.
  - Set $\beta_{t}=\epsilon^{2}$.
  - Update distribution $D_{t}$ as $D_{t+1}\left(i\right)=\frac{D_{t}\left(i\right)}{Z_{t}}\times \left(\begin{array}{cc} \beta_{t} & \mathrm{if}\left|\frac{f_{t}\left(x_{i}\right)-y_{i}}{y_{i}}\right|\leq \phi \\ 1 & \mathrm{otherwise}, \end{array}\right)$ where $Z_{t}$ is a normalization factor chosen such that $D_{t+1}$ will be a distribution.
  - Set t=t+1.
4. Output the final hypothesis: $f\left(x\right)=\frac{\sum_{t}\log\left(1/\beta_{t}\right)f_{t}\left(x\right)}{\sum_{t}\log\left(1/\beta_{t}\right)}$.

In the last algorithm, the usual choice for the weak learner is a regression tree, but it is possible to use another learner.

### 3.5. Gradient Boosting

Gradient Boosting proposed by [42,43], is a powerful ensemble learning technique used for regression and classification tasks, which builds a predictive model by combining multiple (denoted by *M*) weak learners, typically decision trees. The main idea behind Gradient Boosting is to iteratively improve the model’s predictions by focusing on the errors made in previous iterations. The idea is to find a function F(x) that maps x to *y* with the minimum value for a loss function *L*.

The steps for Gradient Boosting are described below.

1. Input:
  - Sequence of *n* examples ${\left\{\left(x_{i},y_{i}\right)\right\}}_{i=1}^{n}$, where $x_{i}\in R^{d}$ represents the input features and $y_{i}\in R$ represents the target values.
2. Initialize the model with a constant value $F_{0}\left(x\right)=\underset{\gamma }{\arg\min}\sum_{i=1}^{n}L\left(y_{i},\gamma \right)$. If we use as loss function $L\left(y_{i},\gamma \right)={\left(y_{i}-{\hat{y}}_{i}\right)}^{2}/2$, then the optimal γ is $\bar{y}$.
3. For m=1 to *M*:
  - Compute the pseudo-residual $r_{im}=-{\left[\frac{\partial L(y_{i},F\left(x_{i}\right))}{\partial F(x_{i})}\right]}_{F\left(x\right)=F_{m-1}\left(x\right)}$ for i=1,2,…,n.
  - Fit a regression tree to the $r_{im}$ values and create terminal regions $R_{jm}$ for $j=1,\ldots ,J_{m}$.
  - For $j=1,\ldots ,J_{m}$ compute $\gamma_{jm}=\underset{\gamma }{\arg\min}\sum_{x_{i}\in R_{ij}}L(y_{i},F_{m-1}\left(x_{i}+\gamma \right)$.
  - Update $F_{m}\left(x\right)=F_{m-1}\left(x\right)+\nu \sum_{j=1}^{J_{m}}\gamma_{jm}I\left(x_{i}\in R_{jm}\right)$. The learning rate ν∈(0,1).
4. Output $F_{M}\left(x\right)$ as the regression function using *M* learners.

### 3.6. Long Short-Term Memory Neural Networks

**Figure 5:**
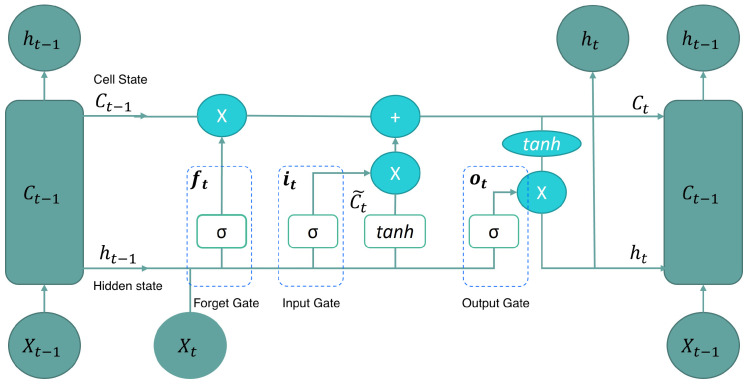
Long short-term memory architecture. Taken from [48].

LSTM architecture consists of four neural networks and specialized memory units called cells. By design, LSTM are capable of preserving information over extended sequences, storing it within these cells. This is achieved through three gates: the forget gate $\left(f_{t}\right)$, the input gate $\left(i_{t}\right)$, and the output gate $\left(o_{t}\right)$. These gates decide what information to add to, remove from, and output from the memory cell. LSTM cell contains two main states: the cell state $\left(C_{\left(t-1\right)}\right)$ and the hidden state $\left(h_{\left(t-1\right)}\right)$, which are continually updated to carry relevant information across time steps. The cell state is responsible for storing long-term memory, while the hidden state holds short-term memory [48]. LSTM structure is illustrated in Figure 5.

#### 3.6.1. Forget Gate

Forget gate decides how much of the previous data will be forgotten and how much of the previous data will be used in next steps. A sigmoid activation function is needed to restrict the information to the range between 0 and 1. The calculation within the forget gate can be performed using:

$$f_{t}=\sigma \left(W_{f}X_{t}+U_{f}h_{\left(t-1\right)}+b_{f}\right)$$

The forget gate receives input from the previous hidden state, $h_{\left(t-1\right)}$, and the current sequence input, $X_{t}$. This data is then combined with a bias term, $b_{f}$, and multiplied by the corresponding weights, $\left(W_{f},U_{f}\right)$. Subsequently, the resulting values pass through the sigmoid activation function, σ, which determines whether the information should be retained in the cell state or discarded.

#### 3.6.2. Input Gate

The input gate collects information from $h_{\left(t-1\right)}$ and $X_{t}$ to update the cell state. This data is processed through two functions: sigmoid and tanh. The sigmoid activation is used to identify which information is important and requires updating. The values from $X_{t}$ and $h_{\left(t-1\right)}$ are combined with a bias term, $b_{i}$, and then multiplied by the respective weights, $W_{i}$ and $U_{i}$, to produce a vector that ranges from zero to one. The sigmoid output is then used to evaluate the relevance of the data using:

$$i_{t}=\sigma \left(W_{i}X_{t}+U_{i}h_{\left(t-1\right)}+b_{i}\right)$$

Next, the equation can be applied to compute the tanh function, which is used to scale the data between −1 and 1 using the following expression. This process generates a new vector that is then applied to update the cell state.

$$C_{t}=\tanh\left(W_{c}X_{t}+U_{c}h_{\left(t-1\right)}+b_{c}\right),$$

where $W_{c}$ and $U_{c}$ represent the weights, and $b_{c}$ denotes the bias.

#### 3.6.3. Output Gate

The process is finalized by incorporating two steps within the output gate. The first step involves obtaining the current input, $X_{t}$, and the previous hidden state, $\left(h_{\left(t-1\right)}\right)$. These values are then passed through the sigmoid function. As with the other gates, the sigmoid activation is applied, after which the results are multiplied by the weights, $W_{o}$ and $U_{o}$, and added to a bias term, $b_{o}$.

$$o_{t}=\sigma \left(W_{o}X_{t}+U_{o}h_{\left(t-1\right)}+b_{o}\right)$$

The next step involves obtaining the value of the cell state and passing it through the tanh activation function. Then, the result of the tanh function is multiplied by the output of the sigmoid function to determine the information for the hidden state [49]:

$$h_{t}=o_{t}\times \tanh\left(C_{t}\right).$$

### 3.7. CC-CNN Method for Preprocessing

**Figure 6:**
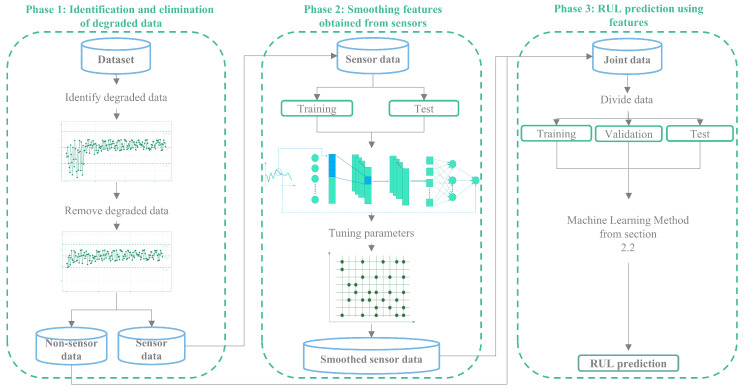
Phases of the preprocessing CC-CNN method.

This section outlines our proposal for preprocessing data obtained from battery data acquisition. Figure 6 illustrates the phases involved in transforming raw data using a CC and a CNN into processed data suitable for input into any machine learning method discussed in Section 3.2. We refer to our proposed method as the CC-CNN method, which consists of three phases.

Handling sensor noise and outliers is critical for ensuring the reliability of predictive models in battery degradation analysis. The first phase involves identifying and eliminating degraded data using individual control charts, as described by [50]. The control chart is constructed by plotting the lower control limit (LCL) and upper control limit (UCL), calculated as follows:

$$\begin{array}{cc} LCL & =\bar{X}-L\sigma \\ UCL & =\bar{X}+L\sigma \end{array}$$

Observations that fall outside the lower control limit (LCL) and upper control limit (UCL) are thresholds which are flagged as potential outliers, indicating significant deviations from the expected range of normal variation. The presence of these degraded data points can be attributed to various factors, including sensor errors, environmental disturbances, or operational anomalies. The elimination of these outliers preserve the intrinsic variability of the degradation process, thereby preventing noise from distorting model training. This methodological approach ensures that predictive models accurately capture meaningful degradation trends rather than being influenced by spurious fluctuations introduced by erroneous measurements.

To enhance the precision of predictions, a time-based criterion is employed to identify failure periods, thereby preventing transient anomalies from distorting long-term trend analysis. This is particularly relevant in real-world conditions, where external factors such as temperature fluctuations and varying load conditions can induce short-term deviations in sensor readings. Then, the dataset is systematically refined to reflect the natural variability of battery degradation through the implementation of statistical filtering and structured data partitioning. This methodological approach enhances RUL estimation ensuring that predictive models rely on valid operational data, enhancing their precision and adaptability. Additionally, to refine predictive accuracy, a time-based criterion is implemented to detect periods of failure, preventing transient anomalies from affecting long-term trend analysis (Figure 6).

In the second phase, we use only the features obtained from sensors, as these measurements can be corrupted due to sensor degradation, calibration errors, electrical noise, software bugs, and other factors. Each feature is smoothed using a CNN, that are a class of artificial neural networks designed to automatically learn spatial hierarchies of features through backpropagation, utilizing key components like convolution layers, pooling layers, and fully connected layers. The essence of CNN lies in their ability to extract features independently, eliminating the need for manual feature extraction methods. This characteristic makes CNN exceptionally efficient in image and sensor processing tasks [51,52]. The central rectangle in Figure 6 represents the second phase. In this phase, the sensor data is divided into training and testing datasets, which are used to train and calibrate the CNN. Finally, using the trained CNN, the sensor data are transformed into smoothed sensor data for use in the next phase.

CNN architecture is characterized by fixed hyperparameters, including a convolutional layer with a filter size of 2 and a ReLU activation function. Additionally, the architecture incorporates a max-pooling layer with a size of 2, a flattening layer, and finally two fully connected layers, with the last dense layer containing a single neuron that corresponds to the smoothing of each variable obtained through the sensors. A CNN model is compiled using the Adam optimizer and the mean squared error (MSE) loss function. This configuration of fixed hyperparameters permitted the optimization of other hyperparameters, including the number of filters, number of neurons, learning rate, and epochs, via grid search. The optimized values obtained were 32, 50, 0.001, and 500, respectively.

The third phase is represented in Figure 6 by the right rectangle. In this phase, we combine the non-sensor data with the smoothed sensor data obtained in the second phase. Once the combined data is consolidated, we divide it into three sets: training, validation, and test. These datasets are the input for any of the machine learning methods presented in Section 3.2.

### 3.8. Measures to Evaluate the Performance

The performance of the models was evaluated based on MSE, RMSE, and Mean Absolute Error (MAE). In this study, $y_{i}$ is the observed value of RUL, and ${\hat{y}}_{i}$ is the predicted value of RUL obtained by the machine learning methods. A smaller value indicates a better RUL prediction. The definitions for these metrics are shown below:

(1) $$\mathrm{MSE}=\frac{1}{n}\sum_{i=1}^{n}{\left(y_{i}-{\hat{y}}_{i}\right)}^{2}.$$

MSE quantifies the deviation between the observed value of the RUL $y_{i}$ and the predicted value of RUL ${\hat{y}}_{i}$ obtained by the machine learning methods. RMSE is the square root of MSE.

(2) $$\mathrm{RMSE}=\sqrt{\frac{1}{n}\sum_{i=1}^{n}{\left(y_{i}-{\hat{y}}_{i}\right)}^{2}}.$$

MAE measures the average absolute difference between the predicted values of RUL and the actual target values.

(3) $$\mathrm{MAE}=\frac{1}{n}\sum_{i=1}^{n}\left|y_{i}-{\hat{y}}_{i}\right|.$$

## 4. Results

In Section 4.1 and Section 4.2, we present the results of the exploratory data analysis and the predictive analysis.

### 4.1. Exploratory Data Analysis

In this section, we present the exploratory data analysis conducted to better understand the collected dataset. The variables selected for inclusion in our analysis are motor voltage, motor current, motor temperature, motor power, battery voltage, battery current, battery temperature, battery power, environmental temperature, controller temperature, and relative humidity. These variables were chosen because they are critical indicators of both motor and battery performance, which directly influence the EVs’ RUL. Certain variables were excluded from the analysis, including the global vibration of the electric vehicle, the angular velocity, the vibration of the controller, the brake status, the barometric pressure, the speed variations, the distance traveled, and the changes in altitude. These variables exhibited minimal variation across all tests; for instance, both barometric pressure and distance traveled remained nearly constant. The lack of variability in these measurements limits their usefulness in predictive modeling. Furthermore, some variables were not considered due to constraints in sensor capture and storage capabilities, which resulted in incomplete data that could compromise the integrity of the analysis.

Table 4 summarizes key statistics for several variables related to the performance and environmental conditions of EVs. Variables include motor and battery voltages, currents, power outputs, and temperature measurements, as well as relative humidity.

The average values for motor voltage (0.4995) and battery voltage (0.4555) are relatively high, indicating that these components are operating close to their normalized maximum levels. The motor current (0.3075) and battery current (0.1438) are significantly lower than their voltage counterparts, suggesting that while voltage levels are high, the current draw remains moderate. The standard deviations across the variables indicate varying degrees of variability in the data. For example, motor current (0.2289) and battery power (0.1255) have higher standard deviations relative to their averages, suggesting that these variables may experience significant fluctuations during operation. The coefficient of variation (CV) provides insight into the relative variability of each variable. Motor current (74.44%) and battery power (78.28%) exhibit high CVs, indicating a substantial degree of variability relative to their means. In contrast, relative humidity (22.73%) and environmental temperature (18.97%) show much lower CV, suggesting more stable conditions in these areas.

Both motor voltage (0.7831) and battery voltage (0.7825) demonstrate strong positive correlations with RUL. This suggests that as voltage levels increase, the RUL of the electric vehicle components also tends to increase, indicating a potential relationship between higher voltages and better performance or longevity. In contrast, motor current (−0.2698) and battery power (−0.2672) show negative correlations with RUL, albeit weaker than the positive correlations observed for voltage. This may imply that higher currents and power outputs are associated with a reduced useful life remaining, possibly due to increased stress on the system. Other variables such as battery temperature (−0.5935), controller temperature (−0.5949), and motor temperature (−0.5918) also exhibit strong negative correlations with RUL, indicating that elevated temperatures in these components could negatively impact their longevity.

Table 5 includes descriptive statistics for the same variables in Table 4 but using the proposed preprocessing method.

The averages for motor voltage (0.4730) and battery voltage (0.4548) are slightly lower than those observed in the previous table without preprocessing. This may indicate adjustments made during preprocessing to normalize or standardize the data. The motor current (0.3098) and the battery current (0.1503) remain consistent with their previous values, suggesting stable operational characteristics. The standard deviations indicate variability within each variable. In particular, the motor voltage has a higher standard deviation (0.2455), suggesting that while the average is lower than before, there is considerable fluctuation in voltage levels during operation. Battery current (0.1021) has a lower standard deviation compared to its average, indicating more stable performance in this area. The coefficient of variation (CV) shows relative variability among the variables. Motor current (69.66%) and battery power (71.74%) exhibit high CVs, indicating significant fluctuations relative to their means. Relative humidity (35.32%) has a lower CV compared to previous measurements, suggesting improved stability under environmental conditions.

Both motor voltage (0.8227) and battery voltage (0.8194) show strong positive correlations with RUL, similar to the previous analysis. This reinforces the notion that higher voltage levels are associated with longer RUL for vehicle components. Motor current (−0.2931) and motor power (−0.2922) maintain negative correlations with RUL, indicating that increased current and power usage may still be linked to reduced longevity. Battery temperature (−0.6453), controller temperature (−0.6809), and motor temperature (−0.6936) exhibit strong negative correlations with RUL, suggesting that higher temperatures in these components could significantly impact their useful life. Battery current (−0.0435) and environmental temperature (−0.0818) show very weak negative correlations with RUL, suggesting minimal impact on RUL.

**Figure 7:**
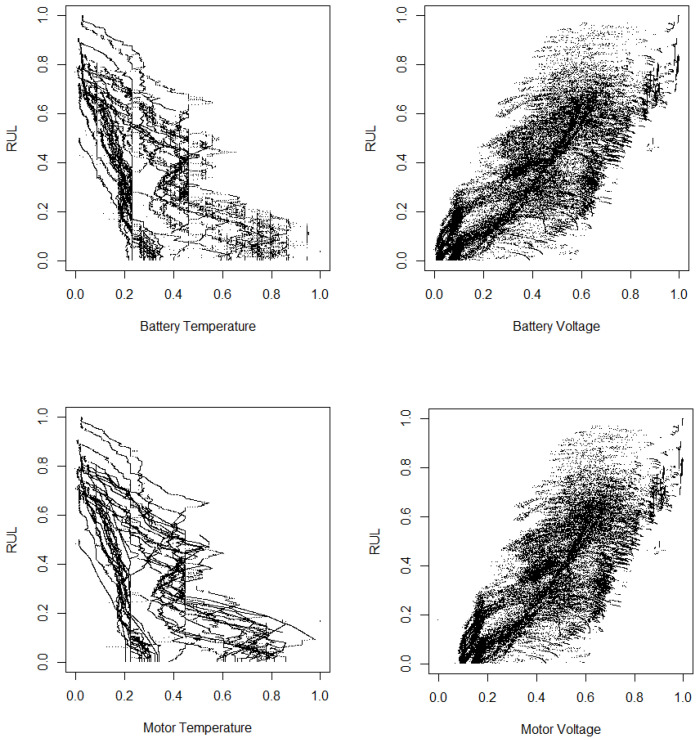
Scatterplot between the RUL and some variables using normalized data without preprocessing.

**Figure 8:**
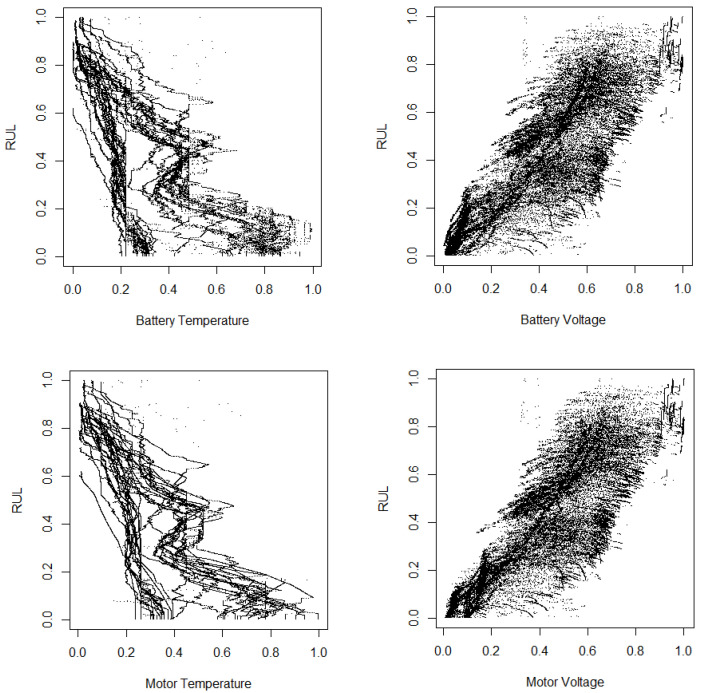
Scatterplot between the RUL and some variables using normalized data with preprocessing CC-CNN.

In Figure 7 and Figure 8, we present scatterplots illustrating the relationships between RUL and selected variables, including battery temperature, battery voltage, motor temperature, and motor voltage. The variables in these figures are normalized to a zero–one interval. As indicated in the previous two tables, there is a positive correlation between RUL and both battery voltage and motor voltage, a finding that is corroborated by the scatterplots. Additionally, the figures confirm the negative correlations between RUL and battery temperature as well as motor temperature. The preprocessing steps applied to the data enhance the correlations between RUL and the explanatory variables. Through careful data cleaning and transformation, we uncover more meaningful relationships and patterns that may have been previously obscured. This improved clarity can lead to more accurate predictions and deeper insights into the factors influencing RUL.

### 4.2. Predictive Analysis

This section presents the results for RUL prediction in EVs using LSTM, SVR, AdaBoost, and Gradient Boost methods. Prediction accuracy is evaluated using standard performance metrics, including RMSE, MAE, and MSE, as defined previously in Section 3.8.

Table 6, Table 7 and Table 8 present performance metrics for the training, testing, and validation datasets, categorized by the type of preprocessing applied and the machine learning methods used. All of these tables show that all performance measures are better when the data is processed with the proposed CC-CNN preprocessing methodology regardless of the machine learning method used.

Table 6 shows that the proposed CC-CNN data preprocessing methodology significantly enhanced the performance of both the SVR and AdaBoost methods. For instance, the RMSE for the SVR method without preprocessing was 301.0560, but with the proposed methodology, it decreased dramatically to 0.0800. This improvement is consistently observed in Table 7 for the testing dataset and in Table 8 for validation dataset.

LSTM method, without data preprocessing, initially showed the best performance across training, test, and validation data (see Table 6, Table 7 and Table 8). Although data preprocessing enhanced LSTM method performance, the improvement was less pronounced compared to SVR and AdaBoost. Gradient Boost achieved the lowest RMSE, MSE, and MAE values when applied to data preprocessed using the proposed CC-CNN methodology, demonstrating superior predictive accuracy.

The reduction in performance metrics means a significant improvement in prediction accuracy, which is critical for real-world electric bicycle applications. Accurate RUL predictions directly influence maintenance planning, enabling proactive intervention rather than reactive repairs. For electric bike users, improved RUL predictions minimize unexpected failures, reduce downtime, and increase reliability for both daily commuting and recreational use. In addition, improved predictive accuracy allows fleet operators to optimize maintenance strategies, avoid premature replacements, and mitigate costly service disruptions. Metrics such as RMSE, MAE, and MSE are quantitative indicators of a model’s ability to minimize prediction errors, essential to building confidence in predictive systems. Inaccurate predictions can lead to either overestimating RUL, resulting in sudden failures, or underestimation, resulting in unnecessary maintenance costs and resource inefficiencies. Therefore, achieving lower RMSE, MAE and MSE values ensures that predictive models provide reliable, actionable insights, ultimately improving user satisfaction and operational efficiency in electric bicycle applications.

**Figure 9:**
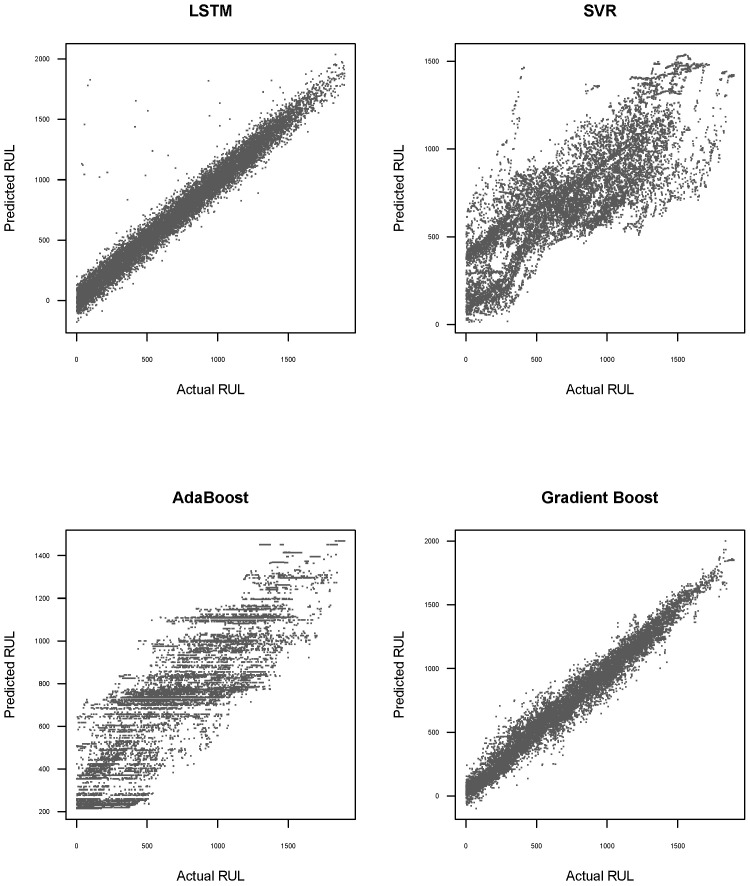
Scatterplot between predicted RUL and real RUL for each method without preprocessing data and using the validation dataset.

**Figure 10:**
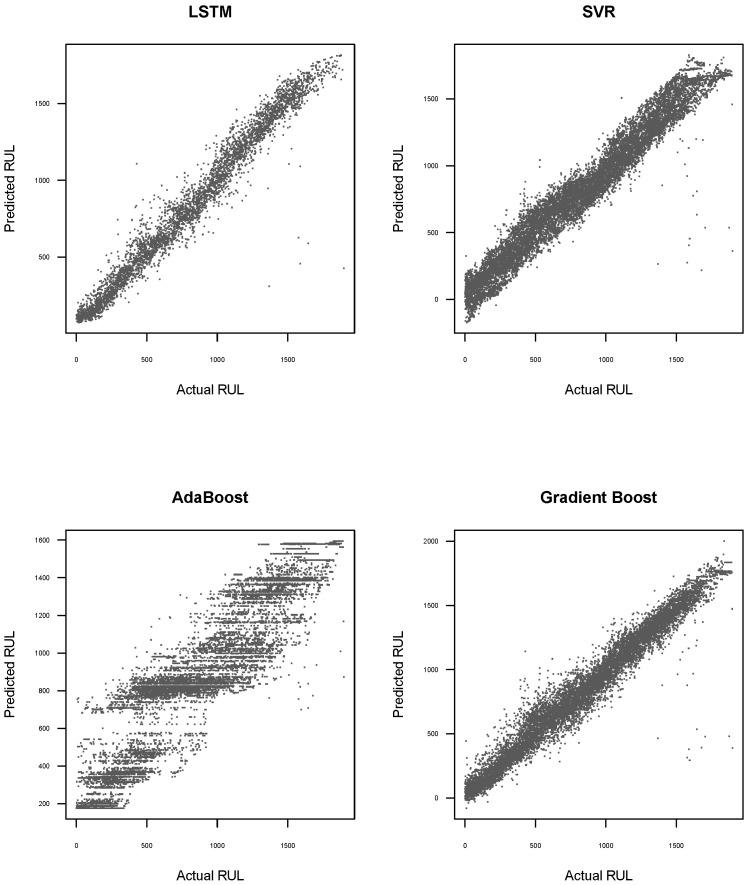
Scatterplot between predicted RUL and real RUL for each method with preprocessing data and using the validation dataset.

In addition to the performance metrics presented above, Figure 9 and Figure 10 illustrate scatterplots that explore the relationship between the predicted RUL for each method and the actual RUL, using the validation dataset. In Figure 9, it is evident that the point clouds for LSTM and Gradient Boost methods exhibit lower dispersion relative to the 45-degree line. This indicates that these methods provide more accurate predictions of the actual RUL. These observations are consistent with the results presented in Table 8.

**Figure 11:**
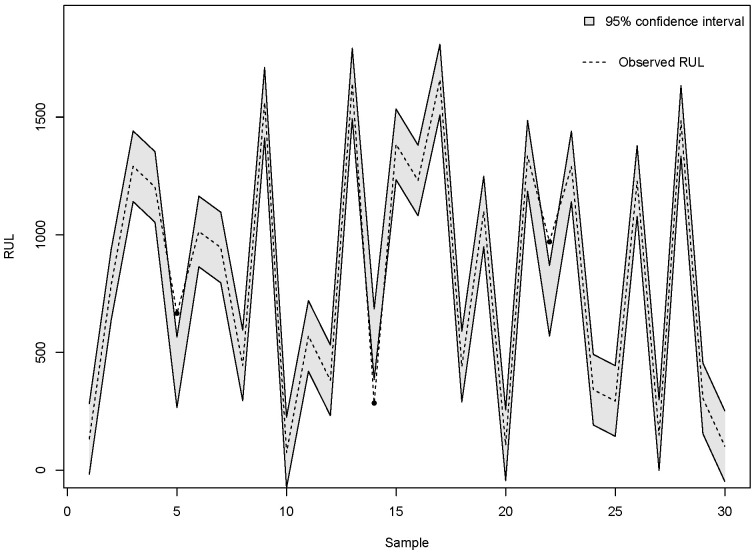
Confidence intervals (95%) for predicting RUL for 30 samples or observations with LSTM method.

Ref. [53] introduced a methodology to assess the SVM uncertainty, which can be adapted to various machine learning techniques. Furthermore, the review by [54] provides a comprehensive overview of predictive uncertainty estimation in machine learning algorithms. Using these methodologies, we constructed a 95% confidence interval, as depicted in Figure 11, to estimate the RUL for 30 selected samples. The results indicate that LSTM model accurately predicts RUL in most instances; specifically, only three cases fall outside the established confidence interval, suggesting a high level of reliability in the model predictions.

## 5. Discussion

The findings of this study demonstrate that the proposed preprocessing method, CC-CNN, significantly enhances the performance of various machine learning algorithms used in the study (LSTM, SVR, AdaBoost, and Gradient Boost) in predicting the RUL of batteries in small EVs. This enhancement highlights the critical role of effective data preprocessing in optimizing predictive models for battery life estimation.

Among the algorithms evaluated, LSTM and Gradient Boost emerged as the most effective techniques, exhibiting superior accuracy and reliability in RUL predictions. These results suggest that integrating advanced preprocessing methods with robust machine learning frameworks for more accurate and actionable BMS insights.

Our results indicate that the best predictive performance was achieved using the Gradient Boosting model, which yielded a RMSE of 0.0270, followed closely by the LSTM model with an RMSE of 0.0489. These findings align with existing literature on RUL prediction. For instance, ref. [26] reported an RMSE of 0.0036 using LSTM combined with Gaussian Process Regression, while [27] achieved an RMSE of 0.0060 through a hybrid approach involving CNN and Gaussian Process Regression. Ref. [28] obtained an RMSE of 46.35 using Random Forest, and [26] also reported an RMSE of 1.096 with the Light Gradient Boosting machine. Additionally, ref. [30] recorded an RMSE of 35.1 using CNN, and [31] achieved an RMSE of 0.0132 with SVR. Ref. [32] reported an RMSE of 0.0274 using AdaBoost, while [33] obtained an RMSE of 0.0136 with Gradient Boosting techniques. Ref. [34] recorded an RMSE of 2.943 using a combination of LSTM and AdaBoost. Ref. [35] achieved an RMSE of 0.0212 with SVR. Ref. [36] reported an RMSE of 117 using CNN-XGBoost. Ref. [11] recorded an RSME of 0.01 using CNN-XGBoost.

Furthermore, the implications of our findings extend beyond small EVs, offering valuable insights that could inform battery life prediction strategies across a range of applications. This research contributes to advancing sustainable energy practices by facilitating more efficient battery management and utilization.

## 6. Conclusions

Based on the results, we can conclude that the proposed CC-CNN data preprocessing methodology enhances the performance of the machine learning methods used in this study for predicting RUL. Moreover, the implications of our findings extend beyond the context of small EVs. The insights gained from this research can inform battery life prediction strategies across various applications, contributing to more informed decision-making in battery management. Ultimately, this work supports the advancement of sustainable energy practices by promoting more efficient utilization and management of battery resources, paving the way for enhanced performance in a range of energy-dependent technologies.

This study not only validates the robustness of the data collected but also underscores its practical importance for sustainable mobility. The results provide key insights for manufacturers to develop more accurate warranty plans, optimized maintenance strategies, and reliable energy management systems for EVs in high altitude and steep terrain environments. In addition, the open-source predictive models developed in this study can be integrated into visualization dashboards that provide real-time battery life estimates to help users optimize charging schedules and energy consumption. These contributions are essential for improving EV reliability and adoption in regions with complex topographies, reinforcing the role of predictive modeling in sustainable transportation systems.

## Figures and Tables

**Table 1:** Previous research on RUL prediction methods.

Methods	Algorithms	Performance Metric	Reference
	LSTM	Relative error = 0.0862	[25]
		RMSE = 0.0036	[26]
		RMSE = 0.0060	[27]
	Random Forest	RMSE = 46.35	[28]
Data-driven	LightGBM	RMSE = 1.096	[29]
methods	CNN	RMSE = 35.1, MAE = 13.7	[30]
		RMSE = 90, MAPE = 10%	[27]
	SVR	RMSE = 0.0132, MAE = 0.0071	[31]
	AdaBoost	RMSE = 0.0274	[32]
	Gradient Boost	RMSE = 0.0136	[33]
	LSTM-AdaBoost	RMSE = 2.943	[34]
Hybrid	CNN-LSTM-ASAN	RMSE = 0.02120, MAE = 0.00896	[35]
methods	CNN-XGBoost	RMSE = 117, MAPE = 12.9%	[36]
		RMSE = 0.01003, MAPE = 0.0059%	[11]

**Table 2:** Average performance under experimental conditions.

Variable	Route
On-Road Time	270 [min]
Distance Traveled	26.89 [km]
Average Speed	24.94 [km/h]
Maximum Speed	38.51 [km/h]
Charging Time	278 [min]

**Table 3:** Variables, sensors, quantity, and precision/tolerance.

Variable	Sensor	Quantity	Precision/Tolerance
Global vibration of EVs	ADXL345	1	±2 g, ±4 g, ±8 g, ±16 g
Angular velocity	Sensor	1	0 to 500 Hz
Controller vibration	SEN-09196	1	0 to 90 Hz
Motor voltage	INA229	4	0.3 V to +85 V
Motor current	INA229	4	0.3 V to +85 V
Battery voltage	INA229	4	0.3 V to +85 V
Battery current	INA229	4	0.3 V to +85 V
Brake	Sensor	1	0 to 500 Fren/seg
Barometric pressure	QMP 6988	1	30 kPa to 110 kPa
Relative humidity	SHT30	1	0% RH to 100% RH
Environmental temperature	SHT30	1	0 °C to 65 °C
Battery temperature	NTCALUG03A103GC	2	−40 °C to +125 °C
Controller temperature	NTCALUG03A103GC	1	−40 °C to +125 °C
Motor temperature	NTCALUG03A103GC	1	−40 °C to +125 °C

**Table 4:** Descriptive measures for some normalized variables without preprocessing.

Variable	Average	Standard Deviation	Coefficient of Variation (%)	Correlation Coefficient with RUL
Motor voltage	0.4995	0.2277	45.5829	0.7831
Motor current	0.3075	0.2289	74.4400	−0.2698
Motor power	0.3067	0.2304	75.1220	−0.2672
Battery voltage	0.4555	0.2480	54.4407	0.7825
Battery current	0.1438	0.1064	74.0209	−0.0506
Battery power	0.1603	0.1255	78.2828	−0.1183
Relative humidity	0.7777	0.1768	22.7333	0.0658
Environmental temperature	0.7384	0.1401	18.9692	−0.0025
Battery temperature	0.3009	0.1992	66.2040	−0.5935
Controller temperature	0.3153	0.2106	66.7818	−0.5949
Motor temperature	0.2923	0.1953	66.7948	−0.5918

**Table 5:** Descriptive measures for some normalized variables with preprocessing CC-CNN.

Variable	Average	Standard Deviation	Coefficient of Variation (%)	Correlation Coefficient with RUL
Motor voltage	0.4730	0.2455	51.9012	0.8227
Motor current	0.3098	0.2158	69.6573	−0.2931
Motor power	0.3110	0.2172	69.8183	−0.2922
Battery voltage	0.4548	0.2549	56.0480	0.8194
Battery current	0.1503	0.1021	67.9380	−0.0435
Battery power	0.1656	0.1188	71.7384	−0.1218
Relative humidity	0.6238	0.2203	35.3197	0.1002
Environmental temperature	0.3239	0.2328	71.8893	−0.0818
Battery temperature	0.3019	0.2124	70.3646	−0.6453
Controller temperature	0.3221	0.2138	66.3662	−0.6809
Motor temperature	0.3107	0.1972	63.4557	−0.6936

**Table 6:** Results for predicting RUL in the training dataset.

Approach	Method	RMSE	MAE	MSE
Without preprocessing	LSTM	0.0715	0.0517	0.0051
	SVR	301.0560	243.3260	90,634.7586
	AdaBoost	195.6430	165.8030	38,279.1262
	Gradient Boost	18.7960	13.2340	353.3660
With CC-CNN	LSTM	0.0455	0.0330	0.0021
	SVR	0.0800	0.0630	0.0600
	AdaBoost	0.1120	0.0920	0.0130
	Gradient Boost	0.0110	0.0070	0.0000

**Table 7:** Results for predicting RUL in the testing dataset.

Approach	Method	RMSE	MAE	MSE
Without preprocessing	LSTM	0.0738	0.0530	0.0054
	SVR	301.0830	243.3640	90,657.8896
	AdaBoost	196.1008	166.0733	38,464.0959
	Gradient Boost	42.9687	26.8464	1847.7117
With CC-CNN	LSTM	0.0481	0.0337	0.0023
	SVR	0.0800	0.0630	0.0600
	AdaBoost	0.1130	0.0920	0.0130
	Gradient Boost	0.0280	0.0150	0.0110

**Table 8:** Results for predicting RUL in the validation dataset.

Approach	Method	RMSE	MAE	MSE
Without preprocessing	LSTM	0.0746	0.0537	0.0055
	SVR	179.2630	118.5989	32,140.5348
	AdaBoost	196.2292	165.7239	38,520.4623
	Gradient Boost	40.3326	25.3439	1628.1875
With CC-CNN	LSTM	0.0489	0.0338	0.0024
	SVR	0.0811	0.0631	0.0066
	AdaBoost	0.1140	0.0929	0.0130
	Gradient Boost	0.0270	0.0143	0.0007

## Data Availability

Data are available upon a reasonable request from the corresponding author.
